# Supplementary material for: Integrated economic and sexual and reproductive health programming among married and unmarried adolescent girls in Nigeria: Results from a quasi-experimental cohort study
Source: PLoS One. 2025 Aug 25;20(8):e0330420. doi: 10.1371/journal.pone.0330420 (PMC12440252; doi:10.1371/journal.pone.0330420)
Supplement: S1 File — (DOCX) [file pone.0330420.s001.docx]

**S1 File: Program exposure questions**

|  | Program Exposure |  |
| --- | --- | --- |
|  | *This block is only to be used at endline for program participants.* |  |
|  | Did you participate in the life mapping / goal setting session? | 1=Yes, 2=No  -98=Refused  -99=Don’t know     - *Closed-ended* - Nominal |
|  | Did you participate in some or all of the Life Family Health (LFH) / Life, Love and Health (LLH)  classes? | 1=Some, 2=All of the sessions  -98=Refused  -99=Don’t know   - *Open-ended, closed coded* - Nominal     *Logic: If the participant is based in Ogun, use Life Love and Health (LLH) and if the participant is based in Kaduna, use Life Family Health (LFH)*    *Note: There are four sessions, so some would encompass 1-3 sessions.* |
|  | As part of the MMA/9ja girls program, there are economic empowerment sessions including topics such as my own pathway, budgeting and savings, business skills, etc.. Did you participate in none, some, or all of these sessions? | 1=None of the sessions  2=Some of the sessions  3= All of the sessions  -98=Refused  -99=Don’t know     - *Closed-ended* - Nominal |
|  | Next, I am going to read you a list of three additional program components. For each one, please indicate ‘yes’ or ‘no’ for whether you participated in that part of the program.     - Vocational skills sessions - Apprenticeship - Market place and graduation - Mentorship | 1=Yes, 2=No  -98=Refused  -99=Don’t know     - *Closed-ended* - Nominal     *Logic: This question is only for those in the “intervention” group.*    *Note: Participants will answer yes or no for each item listed.* |
|  | How many one-on-one coaching sessions did you participate in? | 1=Client answered, 2=Client did not answer    *Logic: If the client provides an answer to this question, the enumerator will select “1” and they will be asked to record a numeric response.* |
|  | How many group coaching sessions did you participate in? | 1=Client answered, 2=Client did not answer    *Logic: If the client provides an answer to this question, the enumerator will select “1” and they will be asked to record a numeric response.* |
