# Supplementary material for: Integrated economic and sexual and reproductive health programming among married and unmarried adolescent girls in Nigeria: Results from a quasi-experimental cohort study
Source: PLoS One. 2025 Aug 25;20(8):e0330420. doi: 10.1371/journal.pone.0330420 (PMC12440252; doi:10.1371/journal.pone.0330420)
Supplement: S2 Table — (DOCX) [file pone.0330420.s003.docx]

| **S3 Table. Comparison of unadjusted and adjusted DiD** | | | | | |  |  |  |  |  |  |  |
| --- | --- | --- | --- | --- | --- | --- | --- | --- | --- | --- | --- | --- |
|  | Unadjusted | | | | | | Adjusted | | | | | |
|  | Kaduna | | | Ogun | | | Kaduna | | | Ogun | | |
| Indicator | DiD | 95% CI | *P*-value | DiD | 95% CI | *P*-value | DiD | 95% CI | *P*-value | DiD | 95% CI | *P*-value |
| Earns Money | 0.36 | 0.281; 0.431 | <0.001 | 0.55 | 0.470; 0.637 | <0.001 | 0.350 | 0.28; 0.43 | <0.001 | 0.58 | 0.50; 0.67 | <0.001 |
| Purchases Assets | 0.08 | 0.008; 0.144 | 0.028 | 0.28 | 0.203; 0.346 | <0.001 | 0.06 | -0.01; 0.13 | 0.103 | 0.31 | 0.23; 0.38 | <0.001 |
| Contributes to Expenses | 0.30 | 0.221; 0.375 | <0.001 | 0.38 | 0.291; 0.465 | <0.001 | 0.29 | 0.21; 0.36 | <0.001 | 0.28 | 0.11; 0.45 | 0.002 |
| Contraceptive Use | 2.31 | 0.860; 3.751 | 0.002 | 0.17 | -0.002; 0.347 | 0.053 | 2.07 | 0.67; 3.48 | 0.004 | 0.18 | -0.01; 0.37 | 0.600 |
| Intent to Use Contraception | -0.01 | -0.147; 0.132 | 0.919 | 0.46 | 0.184; 0.725 | 0.001 | 0.00 | -0.14; 0.14 | 0.891 | 0.48 | 0.21; 0.75 | 0.001 |
