# Supplementary material for: Integrated economic and sexual and reproductive health programming among married and unmarried adolescent girls in Nigeria: Results from a quasi-experimental cohort study
Source: PLoS One. 2025 Aug 25;20(8):e0330420. doi: 10.1371/journal.pone.0330420 (PMC12440252; doi:10.1371/journal.pone.0330420)
Supplement: S3 Table — (DOCX) [file pone.0330420.s004.docx]

**S4 Table**

**Differential attrition analysis table by geography**

**KADUNA**

| **Parameter** | **Category** | **Loss to follow up** | | **Sig.** |
| --- | --- | --- | --- | --- |
|  |  | **Yes** | **No** |  |
| Program Exposure | Comparison Group | 126(60.6%) | 367(43.6%) | **<0.0001** |
|  | Intervention Group | 82(39.4%) | 474(56.4%) |  |
| Purchased an asset | No | 51(24.8%) | 219(26.0%) | 0.7059 |
|  | Yes | 155(75.2%) | 622(74.0%) |  |
| Earn Money | No | 91(43.8%) | 360(42.8%) | 0.8055 |
|  | Yes | 117(56.3%) | 481(57.2%) |  |
| Contribution to Household Expenses | No | 34(29.1%) | 152(31.6%) | 0.5944 |
|  | Yes | 83(70.9%) | 329(68.4%) |  |
| Currently Using contraception | No | 127(62.3%) | 513(62.3%) | 0.9836 |
|  | Yes | 77(37.7%) | 310(37.7%) |  |
| Intend to use contraceptive in future | No | 41(33.3%) | 136(27.1%) | 0.1725 |
|  | Yes | 82(66.7%) | 365(72.9%) |  |
| Age in Years | 15-17 | 39 (18.8%) | 147 (17.5%) | 0.8311 |
|  | 18-19 | 169 (81.3%) | 694 (82.5%) |  |
| Highest level of education | No formal education | 20 (9.6%) | 71 (8.4%) | 0.6170 |
|  | Primary | 24 (11.5%) | 77 (9.2%) |  |
|  | Secondary | 145 (69.7%) | 607 (72.2%) |  |
|  | Above Secondary | 19 (9.1%) | 86(10.2%) |  |
| Number of Children | 0 | 25(12.0%) | 163(19.4%) | 0.0929 |
|  | 1 | 94(45.2%) | 362(43.0%) |  |
|  | ≥2 | 89 (42.8%) | 316 (37.6%) |  |

**OGUN**

| **Parameter** | **Category** | **Loss to follow up** | | **Sig.** |
| --- | --- | --- | --- | --- |
|  |  | **Yes** | **No** |  |
| Program Exposure | Comparison Group | 168(63.9%) | 258(38.9%) | **<0.0001** |
|  | Intervention Group | 95(36.1%) | 406(61.1%) |  |
| Purchased an asset | No | 63(24.0%) | 218(32.9%) | **0.0085** |
|  | Yes | 199(76.0%) | 445(67.1%) |  |
| Earn Money | No | 169(64.3%) | 521(78.5%) | **<0.0001** |
|  | Yes | 94(35.7%) | 143(21.5%) |  |
| Contribution to Household Expenses | No | 51(54.3%) | 83(58.0%) | 0.5651 |
|  | Yes | 43(45.7%) | 60(42.0%) |  |
| Currently Using contraception | No | 32(24.1%) | 51(26.2%) | 0.6685 |
|  | Yes | 101(75.9%) | 144(73.8%) |  |
| Intend to use contraceptive in future | No | 93(74.4%) | 342(73.2%) | 0.7930 |
|  | Yes | 32(25.6%) | 125(26.8%) |  |
| Age in Years | 15-17 | 111 (42.2%) | 376 (56.6%) | **0.0002** |
|  | 18-19 | 152 (57.8%) | 288 (43.4%) |  |
| Highest level of education | No formal education | 4 (1.5%) | 5 (0.8%) | 0.4506 |
|  | Primary | 8 (3.0%) | 15 (2.3%) |  |
|  | Secondary | 237 (90.1%) | 607 (91.4%) |  |
|  | Above Secondary | 14 (5.3%) | 37 (5.6%) |  |
| Number of Children | 0 | 205(77.9%) | 565(85.1%) | **0.0471** |
|  | 1 | 41(15.6%) | 63(9.5%) |  |
|  | ≥2 | 17 (6.5%) | 36 (5.4%) |  |
| Married/Staying as Married | No | 234(89.0%) | 617(92.9%) | **0.0482** |
|  | Yes | 29(11.0%) | 47(7.1%) |  |
