# Supplementary material for: Integrated economic and sexual and reproductive health programming among married and unmarried adolescent girls in Nigeria: Results from a quasi-experimental cohort study
Source: PLoS One. 2025 Aug 25;20(8):e0330420. doi: 10.1371/journal.pone.0330420 (PMC12440252; doi:10.1371/journal.pone.0330420)
Supplement: Attachment — Submitted filename: Responses to Reviewers.docx [file pone.0330420.s007.docx]

January 17, 2025

Dr. Ibrahim Jahun, MD, MSC, PhD
Academic Editor
PLOS ONE

I’m pleased to submit a revised version of our manuscript, “*Integrated economic and sexual and reproductive health programming among married and unmarried adolescent girls in Nigeria: results from a quasi-experimental cohort study*” to be considered for publication in.

We thank the editorial team for this opportunity to submit a revision to our submitted manuscript.

**Responses to the reviewer are provided in line in bold below.**

**Editorial Review**

When submitting your revision, we need you to address these additional requirements.

1. Please ensure that your manuscript meets PLOS ONE's style requirements, including those for file naming. The PLOS ONE style templates can be found at

[https://journals.plos.org/plosone/s/file?id=wjVg/PLOSOne_formatting_sample_main_body.pdf](https://nam11.safelinks.protection.outlook.com/?url=https%3A%2F%2Fjournals.plos.org%2Fplosone%2Fs%2Ffile%3Fid%3DwjVg%2FPLOSOne_formatting_sample_main_body.pdf&data=05%7C02%7Camusau%40psi.org%7C7db551c4c62440439cbe08dd192ed762%7Ccd9cb8ece621472a979a549ab5ba2470%7C0%7C0%7C638694411226485582%7CUnknown%7CTWFpbGZsb3d8eyJFbXB0eU1hcGkiOnRydWUsIlYiOiIwLjAuMDAwMCIsIlAiOiJXaW4zMiIsIkFOIjoiTWFpbCIsIldUIjoyfQ%3D%3D%7C0%7C%7C%7C&sdata=qEObA2%2B%2Bjslw48zEbTZo95iUwuqyWkhteSOVv2K43K4%3D&reserved=0) and 
[https://journals.plos.org/plosone/s/file?id=ba62/PLOSOne_formatting_sample_title_authors_affiliations.pdf](https://nam11.safelinks.protection.outlook.com/?url=https%3A%2F%2Fjournals.plos.org%2Fplosone%2Fs%2Ffile%3Fid%3Dba62%2FPLOSOne_formatting_sample_title_authors_affiliations.pdf&data=05%7C02%7Camusau%40psi.org%7C7db551c4c62440439cbe08dd192ed762%7Ccd9cb8ece621472a979a549ab5ba2470%7C0%7C0%7C638694411226498885%7CUnknown%7CTWFpbGZsb3d8eyJFbXB0eU1hcGkiOnRydWUsIlYiOiIwLjAuMDAwMCIsIlAiOiJXaW4zMiIsIkFOIjoiTWFpbCIsIldUIjoyfQ%3D%3D%7C0%7C%7C%7C&sdata=SuIDKJR27OLfdGTLGObjEYGs0gIEvczXyWXjUhDJkz0%3D&reserved=0)

**The revised manuscript has been formatted aligning with the PLOS ONE style guidance**

2. Please include a complete copy of PLOS’ questionnaire on inclusivity in global research in your revised manuscript. Our policy for research in this area aims to improve transparency in the reporting of research performed outside of researchers’ own country or community. The policy applies to researchers who have travelled to a different country to conduct research, research with Indigenous populations or their lands, and research on cultural artefacts. The questionnaire can also be requested at the journal’s discretion for any other submissions, even if these conditions are not met.

Please find more information on the policy and a link to download a blank copy of the questionnaire here: [https://journals.plos.org/plosone/s/best-practices-in-research-reporting](https://nam11.safelinks.protection.outlook.com/?url=https%3A%2F%2Fjournals.plos.org%2Fplosone%2Fs%2Fbest-practices-in-research-reporting&data=05%7C02%7Camusau%40psi.org%7C7db551c4c62440439cbe08dd192ed762%7Ccd9cb8ece621472a979a549ab5ba2470%7C0%7C0%7C638694411226511420%7CUnknown%7CTWFpbGZsb3d8eyJFbXB0eU1hcGkiOnRydWUsIlYiOiIwLjAuMDAwMCIsIlAiOiJXaW4zMiIsIkFOIjoiTWFpbCIsIldUIjoyfQ%3D%3D%7C0%7C%7C%7C&sdata=oF30s3kNJ57Br9Hy3BKAhJjFskX2TGEgkhY4Qdj2sQU%3D&reserved=0).

Please upload a completed version of your questionnaire as Supporting Information when you resubmit your manuscript.

**The completed inclusivity in global research questionnaire has been submitted as a supporting information file (S7 File)**

3. Please note that funding information should not appear in the Acknowledgments section or other areas of your manuscript. We will only publish funding information present in the Funding Statement section of the online submission form. Please remove any funding-related text from the manuscript.

**This section has been deleted from the revised manuscript**

4. Please include a separate caption for figure 1 in your manuscript.

**The caption for Fig 1 has been included in the revised manuscript**

5. We are unable to open your Supporting Information file [S5 Dataset.dta, S6 Dataset.dta]. Please kindly revise as necessary and re-upload.

**We thank the editorial team for catching this oversight. We have resubmitted the data files as CSV files (S5 File and S6 File) which should be able to open with the Microsoft suite**

**Additional Editor Comments:**

Financial disclosure:

Please add “findings are discussion are the opinions of the authors and do not reflect the position of the sponsors”.

**This has been updated in the submission platform as recommended**

Introduction:

- This section is too long. Even though no word limit provided in author’s guide, there is need to be concise and direct to the points relating to the objectives of the paper. The section now spans to about 5 pages and will be better if the section is concisely reduced to 2.5 pages. This will enable readers not to be lost regarding the study objectives.

**Thank you for this suggestion. We have reduced the introduction section to fit into two and half pages.**

- The section will also benefit from basic statistics about reproductive health indicators among young girls globally and in Nigeria as well as statistics about poverty in this population (when compared to young men counterparts). The section should read like a scientific paper with facts (data) and not like a book.

**We have enriched the introduction with more statistics citing the most recent Multiple Integrated Cluster Surveys of 2021 and the Nigeria Demographic Health Survey 2023/2024 on page 3.**

Methods:

- Recruitment: It is confusing how 400 participants relate to Table 1, whereby 12 groups and 24 upskills were reported. Please make this clearer or reconcile.

**We thank the reviewer for the need to clarify this point. The SRH sessions for both the comparison (SRH only) group and the intervention (SRH and Economic Empowerment) group in both states consisted of a group of 12 adolescent girls. The economic empowerment sessions were nearly twice large in Ogun (sessions of 20 participants each) or equal to two times as large in Kaduna (sessions of 24 participants each). This clarification has been provided in Table 1 on page 8.**

- Sample size: it is unclear how the sample size of 400 was reached. Please elaborate.

**We thank the editor for raising this important point. We estimated the sample size to detect a 10% difference in difference in the primary outcome variable (currently earning money) between the intervention and comparison groups and between the two study time points at the 95% confidence level assuming 50% in both groups were earning money at baseline with 80% power and a 10% attrition rate. This statement has been added in the sub-section on participant recruitment and sampling on page 10.**

- Measures: it will be good to clearly list and define all variables that were collected. Please group these variables into baseline and endline to enable proper comprehension.

**We regret the lack of clarity on the study measures. Both baseline and endline included the same measures. Broadly, the measures were categorized into two domains (a) economic and (b) sexual and reproductive health. There were three measures for the economic domain, and all were binary variables - earning money, purchasing an asset using one's own money and contributing to household expenses. There were two measures for the SRH domain - current use of contraception and intention to use contraception in the future which were also binary variables. We have made revisions on page 11 and 12 to respond to this feedback.**

- Data confidentiality and security: this subsection is missing. Please provide the subsection.

**We have included statements on this on page 13 starting on line 242.**

Results:

- Failure to clearly define variables of interests in the methods makes it difficult to follow and understand the results. There is abundant data and information that should be strategically presented. Consider reducing the length of the results section by referring the readers to appropriate tables for details. Lengthy results make it difficult to follow and link it to discussion and conclusion sections. Also consider using appropriate visuals to show comparison of key findings to enable comparison. This may also reduce the length of the results.

**We regret the confusion the lack of clarity on the study measures affected the ease of following the results. We have addressed this concern in two ways. First, we have specified the measures in the methods section. Second, we have reduced the amount of text in the results section, allowing the reader to engage with the bulk of the results in the tables. We have added Fig 2 and Fig 3 to depict the changes that occurred for the measures in the two domains between baseline and endline. We have added the captions to these figures on page 18**

Discussion and conclusion:

- Please split the section into “Discussion” and “Conclusion and Recommendation”.

**We appreciate this thoughtful suggestion. We have adopted this recommendation in the revised manuscript.**

- Will be good to be concise and to ensure discussion is within the scope and findings of this study.

**This recommendation has been adopted.**

**Reviewers' comments:**

Reviewer's Responses to Questions

**Comments to the Author**

1. Is the manuscript technically sound, and do the data support the conclusions?

The manuscript must describe a technically sound piece of scientific research with data that supports the conclusions. Experiments must have been conducted rigorously, with appropriate controls, replication, and sample sizes. The conclusions must be drawn appropriately based on the data presented.

Reviewer #1: Yes

Reviewer #2: Partly

2. Has the statistical analysis been performed appropriately and rigorously?

Reviewer #1: Yes

Reviewer #2: Yes

3. Have the authors made all data underlying the findings in their manuscript fully available?

The [PLOS Data policy](https://nam11.safelinks.protection.outlook.com/?url=http%3A%2F%2Ftrack.editorialmanager.com%2FCL0%2Fhttp%3A%252F%252Fwww.plosone.org%252Fstatic%252Fpolicies.action%2523sharing%2F1%2F010f01938fbeac6e-1d38ecd3-467b-4b49-b8b4-accc88d1006c-000000%2FrbpMexbDVcw3k-rEFDY16sQ74oDkbBP6pWVHXy48SL0%3D187&data=05%7C02%7Camusau%40psi.org%7C7db551c4c62440439cbe08dd192ed762%7Ccd9cb8ece621472a979a549ab5ba2470%7C0%7C0%7C638694411226523679%7CUnknown%7CTWFpbGZsb3d8eyJFbXB0eU1hcGkiOnRydWUsIlYiOiIwLjAuMDAwMCIsIlAiOiJXaW4zMiIsIkFOIjoiTWFpbCIsIldUIjoyfQ%3D%3D%7C0%7C%7C%7C&sdata=3rPt5EbhaJzfLnVMP4WSei%2BdSKfUtO7Xn6xfPKZ3W7I%3D&reserved=0) requires authors to make all data underlying the findings described in their manuscript fully available without restriction, with rare exception (please refer to the Data Availability Statement in the manuscript PDF file). The data should be provided as part of the manuscript or its supporting information, or deposited to a public repository. For example, in addition to summary statistics, the data points behind means, medians and variance measures should be available. If there are restrictions on publicly sharing data—e.g. participant privacy or use of data from a third party—those must be specified.

Reviewer #1: Yes

Reviewer #2: Yes

4. Is the manuscript presented in an intelligible fashion and written in standard English?

PLOS ONE does not copyedit accepted manuscripts, so the language in submitted articles must be clear, correct, and unambiguous. Any typographical or grammatical errors should be corrected at revision, so please note any specific errors here.

Reviewer #1: Yes

Reviewer #2: Yes

5. Review Comments to the Author

Please use the space provided to explain your answers to the questions above. You may also include additional comments for the author, including concerns about dual publication, research ethics, or publication ethics. (Please upload your review as an attachment if it exceeds 20,000 characters)

Reviewer #1: The study addresses an important gap by examining the effects of integrated economic empowerment and sexual and reproductive health (SRH) interventions on adolescent girls in Nigeria. This is particularly valuable given the unique challenges faced by this demographic.

However there are some improvements I would recommend:

1. **Abstract Clarity**: The abstract is dense, especially in terms of results. It would be beneficial to streamline the findings to highlight the most impactful outcomes.

**We have revised the abstract on page 2 by removing most of the numbers and rewording the results to reduce redundancy.**

2. **Introduction** - Background Context: The background is comprehensive but could benefit from a more focused framing of the economic and SRH challenges specifically faced by adolescent girls in Nigeria. Additionally, contextualizing this intervention in the broader scope of Sub-Saharan Africa would help readers unfamiliar with the region.

**We appreciate this feedback. We have been able to contextualize the background information to the context where the study was conducted and to the sub-Saharan Africa Context on page 3.**

3. Methodological Details:
   • Sampling and Recruitment: More details about the sampling process, including any potential biases, would enhance the transparency and replicability of the study

**We are grateful for this thoughtful suggestion. We have reorganized the participant recruitment sub-section on page 9 and 10 to improve the flow and provided statements to clarify the sampling procedures. Selection bias could have occurred with the intervention group, because mobilisers might have preferred to select girls who were relatives, daughters of their friends or from the same religious group, ethnicity or clan with a motivation to ensure that the intervention reached those closer to them. We conducted a differential analysis comparing baseline characteristics between participants in the intervention and comparison group and found some differences (Table 2). To mitigate the influence of these differences in the analysis models, we included these variables as covariates in the difference-in-difference models. We have included a statement about selection bias under the study limitations on page 27.**

• Covariates and Confounding: The manuscript mentions adjustments for certain covariates. It would be helpful to clarify why specific variables (e.g., age, education) were chosen and to justify their relevance in the analysis.

**Under the sub-section on statistical analysis, we have included a statement on page 14 explaining how the covariates were selected. Briefly, evidence such as that from the synthesis by Stavropoulou M., (2018) identified age, parity and marital status as crucial determinants of the success of economic empowerment programs involving adolescent girls. We also considered variables as covariates if there were baseline group differences for this variable between the intervention and comparison group and this has been cited in the statement we have added.**

4. Discussion and Implications:
   a. Interpretation of Findings: The discussion could benefit from more critical reflection on why certain outcomes varied significantly between Kaduna and Ogun, particularly regarding SRH outcomes. Exploring cultural, social, or structural factors could provide readers with a more nuanced understanding.

**Under the discussion section, we have included statements to illustrate why for some outcomes, intervention effects were detected in Kaduna or Ogun and not in both states such as line 413-416. We believe that differences in the age groups of the participants and marital status might account for the observed differences. Furthermore, our intention was not to compare outcomes between these groups because they are uniquely different and how the interventions were implemented was also contextualized to their life circumstances.**

b. Program Duration and Sustainability: The study was conducted over a nine-month period, which limits long-term outcome assessment. Discussing the sustainability of these outcomes beyond the study period would be valuable.

**We acknowledge that the sustainability of the intervention effects is a challenge with our evaluation. This program was designed to be implemented in a short time to demonstrate the feasibility of implementation and to illustrate potential for showing positive outcomes in the short-term. The program could not be continued for a long term because the investment by the financial partners was not availed. We have cited our inability to show the legacy of these outcomes as a limitation of our study under study limitations on lines 485-489 page 27.**

5. Typographical Errors: A thorough proofreading would help eliminate minor typographical errors and improve readability.

**We regret this error. We have rigorously reviewed the draft to increase accuracy and reduced wordiness to increase readability.**

**Reviewer #2:**

1. Expand the Timeline

The study observed changes over nine months, which might not fully capture the long-term effects of the intervention. Extending the study period would provide more robust insights into sustained outcomes, particularly in economic empowerment and contraceptive use.

**We acknowledge this feedback. The long-term sustainability of the outcomes observed for economic empowerment interventions is a predominant concern for players in this sector. As we have responded to the second reviewer, we have cited the study’s inability to demonstrate the durability of the detected intervention effects as a limitation of our study. Our study was limited to nine months because of lack of financial resources to continue the investment beyond that. We take this as a missed opportunity because it is probable that our intervention contributed to larger and sustained outcomes in the medium and long-term as documented in unpublished data from qualitative interviews conducted with participants, mentors and session facilitators and husbands which affirmed the observed findings through increased involvement income generating activities and transformation of the girl’s decision-making power, agency and self efficacy. This is an important consideration that warrants additional research.**

2. Address Attrition Issues
   • Attrition rates were high, especially in Ogun. Future studies could employ strategies such as incentivizing participation, increasing community engagement, or using mobile follow-ups to reduce loss to follow-up and ensure a more representative sample at endline.

**We appreciate the suggestions about how we could have mitigated the high attrition rates in Ogun. We explored some of these interventions to increase our response rates at the endline such as providing a small incentive for participants after they completed the endline survey, contacted participants through their mobile numbers of an alternative person’s number they had provided during the recruitment period and engaged the mobilisers who had recruited participants to support in physically tracing participants who were unreachable or did not have mobile phones. We also scheduled phone interviews for some participants who had travelled out of the study areas. We noted that recruitment occurred when there was a national university strike in Nigeria and some students were part of the program. The strike was called off midway through the program and students resumed studies. We hypothesize this could have contributed to the high attrition rate. Furthermore, the study sites in Ogun are close to Lagos and migration to the city in search of opportunities is not uncommon. Acknowledging the importance of this information to our readers, we have included statements about how follow-ups were conducted under the data collection section.**

3. Enhance Measurement of SRH Outcomes
   • Include detailed questions on sexual activity (e.g., frequency and recency) to better estimate contraceptive needs among participants. Additionally, qualitative methods like focus group discussions could uncover deeper reasons for changes in SRH behaviors.

**We appreciate this feedback which is aligning with recent calls in the SRH research community of practice for the development and use of person-centered measures to appropriately measure contraceptive need, desire and contraceptive use. This paradigm is only possible if researchers adequately include age-appropriate and validated questions in survey tools investigating need, desire and use. In our study, we attempted to be responsive by including a question to determine presence of sexual activity in the past 12 months and excluded girls who were not sexually active from the analysis for current contraceptive use. In hindsight, we could have included a few additional questions to enable us to determine the congruence between participants’ desire and the state of contraceptive use which is more person-centered than contraceptive use as we had done in a recent study in northern Nigeria by Rothschild et al., (2024) -** [**Preference-aligned fertility management among married adolescent girls in Northern Nigeria: assessing a new measure of contraceptive autonomy | BMJ Global Health**](https://gh.bmj.com/content/9/5/e013902)**. This is a missed opportunity for our current manuscript. We also want to acknowledge the precarious circumstance we found ourselves attempting to strike a balance between comprehensive measurement across the two outcome domains cited in our article and the desire to avert participant fatigue. The proposed suggestion warrants detailed investigation in future research.**

4. Explore Regional Variability
   • The study noted significant differences in outcomes between Kaduna and Ogun. Future research should delve into the contextual factors, such as cultural norms and economic conditions, to tailor interventions more effectively for each region.

**Thank you for this important feedback. The interventions tested in our study were designed with this in mind. The contexts in northern Nigeria and southern Nigeria are very different and the study participants in the two contexts were also distinct in terms of age, marital status, and other factors. The intention of our effort was to demonstrate that if we design interventions meeting the needs, aspirations and expectations and fit for purpose within the contexts they will be implemented, the outcomes will be most promising. Our study has demonstrated that these uniquely designed interventions deliver promising outcomes in the short-term. However, outcomes were not detected for all intended outcomes, implying that additional research is required to determine the specific issues within the interventions or within the contextual which might need to be addressed to optimise the interventions for better outcomes holistically. We have acknowledged our study’s limitation and call upon researchers to explore the issues we have cited as gaps in future research.**

5. Incorporate Broader Indicators of Empowerment
   • While economic empowerment was assessed through earnings, asset purchases, and contributions to household expenses, consider adding measures like financial independence, agency in decision-making, and changes in social norms as broader indicators.

**We sincerely appreciate this feedback. We content that economic empowerment outcomes yield outcomes across multiple aspects of the participants lives. Although we collected data to determine the interventions effectiveness on agency outcomes (i.e., self efficacy, decision making and mobility permission) we decided to restrict this paper to the economic and SRH outcomes recognising the need to avoid crowding the article and to be able to thoroughly discuss these outcomes. We have developed a separate manuscript that delves into the interventions’ outcomes on participants’ measures of agency. This draft manuscript is undergoing peer review in a different journal.**

6. Strengthen Comparisons
   • In non-randomized designs, controlling for potential confounding variables is critical. Future studies could implement propensity score matching or more sophisticated econometric techniques to reduce bias in comparisons between intervention and control groups.

**This is a welcome suggestion. We included four covariates – age, parity, marital status (for Ogun only) and highest level of education in our analysis to control for confounding. These were chosen because literature has associated them with economic and SRH outcomes. We believe that there are more factors that our study could have employed in the models to further control confounding. Given the limitations of the variables available for us and the sample size limitations, we agree with this suggestion that future research should purpose to employ sophisticated econometric techniques to control for confounding in the effectiveness models.**

7. Consider Mixed Methods
   • Quantitative surveys provide key outcome measures, but qualitative data can enrich the understanding of barriers, motivators, and participants' perceptions. Adding interviews or focus groups could offer deeper insights into program impacts.

**We appreciate this feedback. Recognising the need to gather additional data to triangulate the study results and to contextualise the observations made, we conducted in-depth interviews with participants in the intervention group and key informant interviews with the session facilitators and mentors. This data was useful in affirming the findings contained in this manuscript and provided a nuanced understanding of the implementation barriers and facilitators. We are in the final process of developing a separate manuscript covering the qualitative findings which will also be available to our readers who might be interested to appreciate the implementation exigencies and the levers for improved implementation outcomes.**

8. Diversify Economic Empowerment Activities
   • Tailor vocational training to include more high-demand, scalable skills, and expand mentorship duration to ensure participants gain practical, sustainable income-generating capabilities.

**This is a welcome suggestion. We agree that experts should invest in designing vocational and entrepreneurial training that matches the target participants’ needs, preference and aspirations but which are also cognisant of the market value of the gained skills. The design of the interventions in our study employed human-centered design (HCD) techniques and involved adolescent girls and community members in the design process. This process is described in an article by Cutherell et al (2023) -** [**https://gatesopenresearch.org/articles/7-106/v1**](https://gatesopenresearch.org/articles/7-106/v1)**. Briefly, the HCD approach was adopted to ensure that program content, delivery approach, program duration were aligning with what girls and their key influencers wanted. Following this process, we observed that girls wanted a blend of traditional skills (hair dressing, bead making, tailoring etc) and modern skills (computer skills, automobile etc). Our intervention facilitated girls with a blend of these skills and girls were allowed to choose the skills that they preferred. As we have cited earlier, our intervention duration was constrained by financial resources and agree that future programs should focus on understanding which of the gained skills contributes to larger and sustained intervention effects and determining the optimal duration of mentorship to ensure that the cost-effectiveness of the interventions is optimised.**

9. Engage Influencers More Effectively
   • The study showed promising results from engaging community leaders. Future interventions could expand this engagement to include more extensive workshops for parents, husbands, and community gatekeepers to amplify support for girls' empowerment.

**We appreciate this feedback. Our interventions included a key influencer engagement component involving community leaders and the girls’ immediate relatives – mothers in Ogun and husbands in Kaduna. This effort was crucial because of a need to create an enabling environment for girls to engage in the training but also to find an amiable space for pursuing their economic aspirations. This was crucial because in this context, women’s role in contributing to the household’s financial security and in engaging in income generating activities is constrained by inequitable norms. Engaging girls’ influencers contributed to the intervention outcomes as confirmed by the qualitative research we conducted. We will be keen to ensure the evidence we gathered on the importance of involving the girls’ key influencers is clearly articulated when we package the findings in the manuscript.**

10. Consider Scalability
    • Evaluate cost-effectiveness and scalability of the combined intervention, ensuring that successful elements can be adapted and expanded to other regions or country

**We acknowledge this suggestion. The primary purpose of our study was to develop a proof of concept on girl-centered economic empowerment interventions for the Nigeria context and to generate learning about whether they work or not. We concur that evidence on the cost-effectiveness of these interventions and their potential for scalability would be useful to inform governments, funders and implementers in making decisions about the adoption and scale-up of these interventions. We regret that these two components were not included in our study. We recommend that future research should invest to gather evidence on these two crucial aspects.**

We thank the editor for this opportunity to submit a revision and would be very happy to address any addition questions or suggestions from the editor or reviewer(s).

Sincerely,

Mary Philips
